# Immunocompromised Status Definition in Observational Studies Using Electronic Health Records: A Scoping Review and a Proposal for a Phenotype Identification Algorithm

**DOI:** 10.1002/pds.70362

**Published:** 2026-03-27

**Authors:** Judit Riera‐Arnau, Nicoletta Luxi, Fabio Riefolo, Martín Solorzano, Irene Pazos, Elena Ballarín, Lise Skovgaard Svingel, Lorenzo Chiusaroli, Elisa Martín‐Merino, Elisa Barbieri, María Lopez‐Lasanta, Sima Mohammadi, Denis Rotta, Alexandra Pacurariu, Catherine Cohet, Miriam Sturkenboom, Carlos E. Durán, the SAFETY‐VAC study consortium, Carlos E. Durán, Miriam Sturkenboom, Judit Riera‐Arnau, Nicoletta Luxi, Olaf Klungel, Patrick Souverein, Sima Mohammadi, Fabio Riefolo, Irene Pazos, Rosa Gini, Davide Messina, Giuseppe Roberto, Carlo Giaquinto, Elisa Barbieri, Luca Stona, Felipe Villalobos, Martín Solorzano, Carlo Alberto Bissacco, Antonio Gimeno, Beatriz Poblador, Mercedes Aza, Aida Moreno, Alejandro Santos, Vera Ehrenstein, Lise Skovgaard Svingel, Benjamin Randeris Johannesen, Cécile Droz‐Perroteau, Laure Carcaillon‐Bentata, Anna‐Mija Tolppanen, Sirpa Hartikainen, Thuan Vo, Anne Paakinaho, Blair Rajamaki, Hedvig Nordeng, Saeed Hayati, Mahmoud Zidan, Juan José Carreras Martínez, Arantxa Urchueguía Fornes, Elisa Correcher Martínez, Javier Díez‐Domingo, Mar Martin, Patricia Garcia‐Poza, Airam de Burgos, Belén Castillo‐Cano, Elisa Martín‐Merino

**Affiliations:** ^1^ Department of Data Science and Biostatistics, Julius Center for Health Sciences and Primary Care University Medical Center Utrecht (UMCU) Utrecht the Netherlands; ^2^ Department of Clinical Pharmacology, Vall Hebron Institut de Recerca (VHIR) Universitat Autònoma de Barcelona (UAB) Barcelona Spain; ^3^ Department of Medicine University of Verona Verona Italy; ^4^ Partnerships, Barcelona Health Hub Teamit Institute Barcelona Spain; ^5^ Fundació Institut Universitari per a la Recerca a l'Atenció Primària de Salut Jordi Gol i Gurina (IDIAPJGol) Barcelona Spain; ^6^ Department of Clinical Epidemiology and Department of Clinical Medicine Aarhus University and Aarhus University Hospital Aarhus Denmark; ^7^ Division of Pediatric Infectious Disease, Department for Women's and Children' Health University of Padua Padua Italy; ^8^ Spanish Agency of Medicines and Medical Devices‐AEMPS Madrid Spain; ^9^ European Medicines Agency (EMA) Amsterdam the Netherlands; ^10^ Vaccine Monitoring Collaboration for Europe (VAC4EU) Brussels Belgium

**Keywords:** diagnostic code, immunocompromised, immunodeficiency, immunosuppressant, phenotype, real‐world data

## Abstract

Immunocompromised individuals experience an impaired immune function due to conditions that might be either congenital or acquired over the course of their lives. Epidemiological studies often rely on clinical definitions which, in some cases, benefit from being translated into machine‐readable algorithms for application to electronic health records (EHRs) databases. The transient nature of certain immunocompromised states and the variability of phenotypes, definitions, coding practices, and data availability entangle this operation. To address these challenges, we conducted a scoping review of existing immunocompromised status definitions in MEDLINE, focusing on epidemiologic and pharmacoepidemiologic studies involving immunocompromised populations. Data extraction was guided by clinical experts, categorizing conditions and medications into seven categories: genetic/hereditary conditions, infectious diseases, malignancies and chemotherapy, organ and stem‐cell transplantations, severe systemic conditions, immunosuppressive drugs, and autoimmune conditions associated with immunosuppressant use. Out of 137 citations, 56 studies were included. Most of the studies focused on a particular disease or therapeutic area. Frequently cited diagnoses included HIV/AIDS (17.9%) and organ transplantation (14.2%). Methotrexate, corticosteroids, TNF‐alpha inhibitors, and calcineurin inhibitors were the most common drugs used to define immunocompromised status. Building on this review and expert opinion, we developed a phenotype algorithm that combines diagnostic, therapeutic, and procedural data in a modular way to identify immunocompromised populations in EHR data sources. The proposed phenotype algorithm can be applied across diverse data sources, settings and research questions. Future research should test its applicability across heterogeneous EHR data sources.

## Key Points

- A proper phenotype algorithm to identify immunocompromised individuals is crucial in epidemiologic and pharmacoepidemiologic research.
- This is the first systematic attempt to describe the operational definitions to identify immunocompromised populations in studies using electronic healthcare records data sources.
- Based on the review results and expert opinion, we developed a phenotype algorithm to identify immunocompromised individuals when conducting research using EHR data sources. Several clinical conditions, medicines, and laboratory tests were joined using sequential logic.
- A challenge when attempting to identify individuals with immunocompromised status is the dynamic nature of secondary immunodeficiencies. The proposed phenotype algorithm partially addresses this challenge by considering the time‐period most likely to define the immunocompromised status.

## Plain Language Summary

Immunocompromised individuals have weakened immune systems due to a congenital condition, an illness acquired later in life, or certain medical treatments. Some people might be immunocompromised only for a short period of time, and the variability of symptoms and diagnoses can vary widely. Hence, the identification of immunocompromised individuals in electronic healthcare records (EHRs) poses several challenges. We reviewed published studies to understand how other researchers have previously identified immunocompromised patients in EHR to conduct pharmacoepidemiologic studies. We grouped medical conditions and medications into seven categories: genetic conditions, infections, cancer and cancer treatments, organ and stem‐cell transplants, serious systemic illnesses, immunosuppressive drugs, and certain autoimmune diseases. In total, we reviewed 56 studies. The most frequently used diagnoses to identify immunocompromised individuals were HIV/AIDS and organ transplantation. Commonly used medications included methotrexate, corticosteroids, and other drugs that suppress the immune system. Building on this review and expert opinion, we developed an algorithm that combines diagnostic codes, medicines, and medical procedures to help identify immunocompromised populations in EHR data sources, facilitating the generation of more robust evidence in future studies.

## 1 Introduction

Immunocompromised individuals, defined as those unable to mount an adequate immune response represent a heterogeneous population with various types and degrees of immunodeficiency affecting humoral and/or cellular immunity [1, 2]. An individual's immunocompromised status may stem from primary immunodeficiency diseases (PIDs), such as genetic or hereditary conditions intrinsic to the immune system, for example, severe combined immunodeficiency (SCID), or from secondary immunodeficiency diseases (SIDs), acquired over the life course, such as human immunodeficiency virus (HIV) infection, organ transplantation, or hematological malignancies. Furthermore, exposure to immunosuppressants or immunosuppressive drugs like systemic corticosteroids or chemotherapy can also contribute to a compromised immune status [3]. The percentage of immunocompromised individuals in the general European population has been reported to vary between 1.4% and 5.4% [4, 5]. Immunosuppression has been associated with both increased hospitalization rates [6, 7, 8, 9] and an increased mortality risk due to intensive care unit (ICU) ventilator‐associated pneumonia compared with non‐immunocompromised individuals (64% vs. 34%), mainly due to multidrug‐resistant pathogens [10].

Applying up‐to‐date definitions and criteria to identify immunocompromised individuals in healthcare databases may reduce the risk of population misclassification and therefore, improve outcome assessment and interpretation of effectiveness and safety studies of medicines, including biologicals and vaccines [4]. As non‐interventional studies rely on clinical definitions, these must be translated into machine‐readable algorithms to identify immunocompromised individuals in electronic health records (EHRs) data sources effectively. The main challenges arise from the high phenotypic variability of the conditions leading to an immunocompromised state, and the temporary status of these conditions in secondary immunodeficiencies. For instance, an individual with leukemia may no longer be immunocompromised once their disease enters remission and leukocyte counts recover. Similarly, a person receiving biologic immunosuppressive therapy may no longer be immunocompromised once the therapy is completed and enough time for immune recovery has elapsed.

This work aimed to describe the existing operational definitions for identifying immunocompromised individuals in epidemiological studies using healthcare databases, and to subsequently propose a phenotype algorithm for their identification in EHR databases.

## 2 Materials and Methods

### 2.1 Study Design, Information Sources, and Search Strategy

A scoping literature search was conducted to identify studies in the MEDLINE database. The studied period ran from inception to August 15, 2024 (data lock point). Our search string included key terms related to the following main concepts: (i) observational studies using healthcare databases, (ii) immunocompromised host, immunosuppression, and immunosuppressants, and (iii) coding systems, see Table S1. We followed the Preferred Reporting Items for Scoping Reviews (PRISMA‐ScR) guidelines [11]. All titles and abstracts were independently screened by six reviewers and any disagreements were resolved by consensus. The final set of full‐text studies was split into six subsets, and the relevant information was extracted independently by six reviewers (J.R.‐A., C.D.S., N.L., M.S., I.P., and F.R.). Each reviewer was paired with another reviewer who cross‐checked the extracted information. Discrepancies were resolved through discussion and mutual agreement among reviewers in two reconciliation sessions.

### 2.2 Eligibility Criteria

We included titles and abstracts of non‐interventional studies using EHR and administrative databases focusing on immunosuppressants, immunocompromising conditions, and immunocompromised populations. Then, the full‐text reading led to the final manuscript selection based on the availability of definitions and diagnostic or drug codes used to retrieve immunocompromised individuals. We excluded interventional studies and studies with primary data collection, case reports, and studies that did not use human health data. Reviews, letters, protocols, editorials, conference abstracts, and non‐peer‐reviewed articles were also excluded. We did not apply any time (except for the end date) or language restriction.

### 2.3 Data Charting Process

The extraction of information from full‐text articles was conducted using a standardized data abstraction tool created in RedCap (v 14.1.0), a customizable informatics systems‐based web software. Main items included: (i) identification of the study (first author, year, digital object identifier [DOI], and title), (ii) study design (cross‐sectional, cohort, case‐only, case–control, other, or not reported. If more than one design was used, the one used to answer the main question was selected), (iii) main study topic (safety, effectiveness, drug utilization, coding system validation, algorithm validation, or epidemiology descriptive or/and association), and (iv) study size and number of included data sources, (v) clinical definitions as plain text or diagnosis codes using a prespecified dictionary, (vi) therapeutic groups or individual drugs used to define immunocompromised populations (drugs were mapped to the 2024 version of the Anatomical, Therapeutic and Chemical [ATC]/Defined Daily Dose [DDD] classification from the World Health Organization [WHO]) [12], and (vii) algorithms (combinations of criteria) used to operationalize the identification of immunocompromised populations. As the focus of this work was not on the results of the studies themselves but on the methods applied to identify immunocompromised populations, a formal quality assessment of the selected studies was not considered relevant for the final selection of studies for inclusion, and was therefore not performed.

### 2.4 Synthesis of Results

Standard descriptive analyses were performed, including counts and percentages. All data analyses were conducted using R (v 4.4.1). Once a set of medical conditions (diagnostic codes), medicines (ATC codes) and algorithms were extracted from the selected studies, two members of the core study team (C.E.D. and F.R.) proposed a set of categories to group the identified conditions and medicines. Four clinical specialists (L.C.H., L.S.S., M.L.L., and D.R.) from Padova University Hospital, Italy, Aarhus University Hospital, Denmark, Vall d'Hebron University Hospital, Spain, and the Rheumatology Unit of the University of Verona, Italy, provided input on the proposed categories, the assignment of medical conditions and medicines to the categories, and recommended exclusion or inclusion of additional conditions or drugs not identified in the literature search.

### 2.5 Development of a Phenotype Algorithm to Identify Immunocompromised Individuals

Based on the set of medical conditions, medicines and algorithms extracted and curated by clinical specialists, two clinicians‐epidemiologists from the core study team (J.R.‐A. and E.B.) developed the identification algorithm. They adjusted and joined the proposed concepts using Boolean terms and logical sequences. In addition to the medical conditions and the medicines, information on diagnostic tests, medicine dosages, and duration of exposure was incorporated when needed. Moreover, in the phenotype algorithm section dedicated to children < 1 year of age, additional conditions to identify bacterial, viral, fungal, and opportunistic infections were added by the core team to improve the accuracy of the definition. Finally, three core team members (C.E.D., F.R., and I.P.) and four clinical specialists (L.C.H., L.S.S., M.L.L., D.R.) reviewed the proposed phenotype algorithm.

Finally, identified clinical conditions were mapped across different medical dictionaries using the CodeMapper tool [13], a semiautomatic web application that assists in mapping case definitions to codes from different vocabularies. CodeMapper generated independent code lists per clinical condition. Each code list underwent independent review by two clinical reviewers, with discrepancies resolved by a third reviewer to reach consensus.

## 3 Results

### 3.1 Characteristics of the Included Studies

**FIGURE 1:**
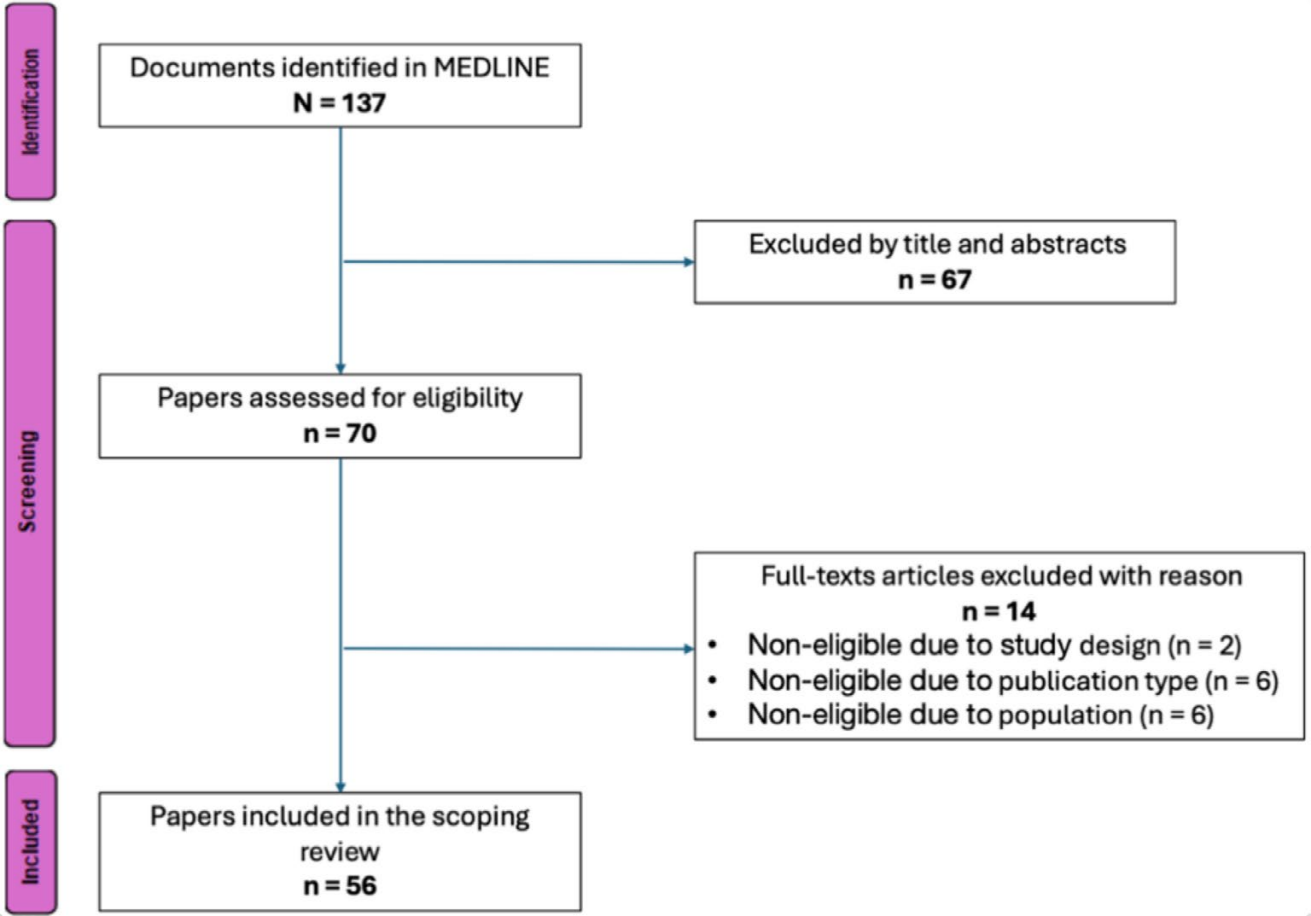
PRISMA diagram of article selection.

Out of 137 citations identified from the search in MEDLINE, 67 were initially excluded after title and abstract review. Seventy studies were assessed in full, out of which 56 were finally selected for data extraction, see Figure 1 and Table S2.

Twenty‐two (39.2%) of the studies were descriptive epidemiologic studies, 25.0% (*n* = 14) explored safety outcomes, 17.8% (*n* = 10) applied an association design, 10.7% (*n* = 6) were drug utilization studies, and algorithm validation and effectiveness studies were 3.5% (*n* = 2) each. A summary of the key characteristics of the included studies is presented in Table 1.

**TABLE 1:** Summary of key characteristics of the studies included in the scoping review.

Key characteristics	Total (*n* = 56)
Number of articles by year of publication, *n* (%)	56 (100)
Before 2015	9 (16.1)
2015–2020	32 (57.1)
After 2020	15 (26.8)
Main study topic, *n* (%)	56 (100)
Safety	14 (25.0)
Effectiveness	2 (3.5)
Epidemiology, descriptive	22 (39.2)
Epidemiology, association	10 (17.8)
Drug utilization	6 (10.7)
Algorithm validation	2 (3.5)
Population being studied, *n* (%)^b^	78 (100)
Adults	40 (51.3)
Paediatrics	8 (10.3)
Elders	17 (21.8)
Pregnant women	2 (2.6)
Any population or no specific exclusions reported	11 (14.1)
Number of databases involved, median [range]	1 [1–6]
Type of database^b^, *n* (%)	73 (100)
Administrative or claims	39 (53.4)
EHR or EMR	18 (24.6)
Registries^a^	8 (11.0)
More than one type^a^	6 (8.2)
Concepts used to define immunocompromised status^b^, *n* (%)	126 (100)
Clinical conditions (diagnostic codes)	51 (91.1)
Clinical specifications (free text)	44 (78.6)
Medicines´ use (ATC codes)	24 (42.8)
Algorithms	7 (12.5)

Abbreviations: ATC, Anatomical Therapeutic Chemical classification; EHR, electronic healthcare records; EMR, electronic medical records.

^a^ Registries included population registries (such as research patient registries, intensive care unit registries, or perinatal registries), disease registries (such as transplant or HIV registries), or registries with specific aims (like the Rochester Epidemiology Project).

^b^ For these items, the sum of percentages is over 100% as more than one category may apply per study.

### 3.2 Identification of Immunocompromised Populations in the Literature

Out of the 56 selected studies, 27 (48.2%) primarily focused on immunocompromised individuals, while 29 (51.8%) only included these populations in specific subgroups or analyses, without making them the main focus of the study. None of the studies aimed to capture an immunocompromised population as a whole; rather, each study was centered on specific factors leading to an immunocompromised state. The studies applied different strategies to identify immunocompromised populations: 51 (91.1%) of the studies used clinical conditions (diagnostic codes), 44 (78.6%) used clinical specifications as free text, 24 (42.8%) used medicines (ATC codes), and seven (12.5%) used algorithms (see Table 1). Of the studies that used clinical conditions and drugs to identify the immunocompromised populations, most used administrative data, followed by EHR and population registries. In addition, 16 used previously validated codes or algorithms (see Table S2). The diagnostic codes most frequently cited were HIV infection‐ or AIDS‐related codes, accounting for 10 of the 56 selected studies (17.9%), followed by codes related to solid organ transplantation status or rejection in eight studies (14.3%) (see Table S3). Drug codes for methotrexate, corticosteroids, selective immunosuppressants, TNF‐alpha inhibitors, and calcineurin inhibitors were the most frequently retrieved. Finally, seven studies (12.5%) applied an algorithm to detect immunocompromised populations, combining diagnostic codes with requirements for prescriptions, analytical and/or imaging tests, number of healthcare visits, and other variables [11, 14, 15, 16, 17, 18, 19], see Table 2.

**TABLE 2:** Algorithms to identify immunocompromised populations in the literature.

First author and year of publication	Manuscript title	Algorithm
Su C.‐F. (2021) [15]	Epidemiology and risk of invasive fungal infections in systemic lupus erythematosus: a nationwide population‐based cohort study.	Invasive fungal infections diagnoses were further validated by a record of in‐hospital prescriptions of systemic antifungal agents available in Taiwan, namely fluconazole, itraconazole, posaconazole, voriconazole, amphotericin B, liposomal amphotericin B, caspofungin, micafungin, anidulafungin, and flucytosine.
Goldberg D. (2016) [16]	Patients with hepatocellular carcinoma have highest rates of wait‐listing for liver transplantation among patients with end‐stage liver disease.	Significant liver disease (SLD) was defined using algorithms based on ICD‐9‐CM codes that have been validated to have positive predictive values of > 85%. All patients first required a diagnosis of cirrhosis: ≥ 1 inpatient or ≥ 2 outpatient ICD‐9‐CM codes for cirrhosis (571.2, 571.5). Decompensated cirrhosis was then defined by having ≥ 1 inpatient or ≥ 2 outpatient ICD‐9‐CM codes for a complication of portal hypertension (ascites, bleeding esophageal varices, and/or spontaneous bacterial peritonitis) occurring after the diagnosis of cirrhosis. HCC required a diagnosis of cirrhosis and ≥ 1 inpatient or ≥ 2 outpatient ICD‐9‐CM code for HCC (ICD‐9‐CM code, 155.0). In the subset of patients with cirrhosis without HCC or a complication of portal hypertension, laboratory criteria was used to define hepatocellular dysfunction (calculated MELD score ≥ 15 and/or a total serum bilirubin ≥ 3 mg/dL; only available for *n* = 3499, 20.8% of the HealthCore cohort; available laboratory data were based on capitation to a specific laboratory and not any particular demographic). The age cutoff for inclusion was 18–75 years at ESLD diagnosis. Patients were excluded if they had an extrahepatic malignancy, excluding nonmelanoma skin cancer, diagnosed within 365 days before the ESLD index date.
Joly M. (2023) [17]	Progressive multifocal leukoencephalopathy: Epidemiology and spectrum of predisposing conditions.	To assess the reliability of progressive multifocal leukoencephalopathy (PML) diagnosis code (A81.2 “Multifocal leukoencephalopathy,” under A81 “Atypical viral infections of the CNS” in the ICD‐10), this algorithm considered that a patient had incident PML if he/she met the following criteria: (i) presence of the A81.2 code as primary diagnosis (PD) code and/or optional related diagnosis (RD) in the public and private hospital discharge database (PMSI); (ii) presence of a predisposing immunosuppressive condition ICD‐10 code (including HIV infection, hematological malignancy, chronic inflammatory disease, solid neoplasm, solid organ transplantation [SOT], and primary immune deficiency [PID]) in the PMSI from 2 years before to 1 year following PML diagnosis; (iii) presence of a brain MRI within 6 months before PML diagnosis and in order to select patients with incident PML; and (iv) absence of the A81.2 code in the PMSI before validation study start date.
Lee W.J. (2018) [18]	Risk of serious bacterial infection associated with tumor necrosis factor‐alpha inhibitors in children and young adults with inflammatory bowel disease.	We identified patients age < 30 years diagnosed with IBD (ICD‐9 code 555.xx or 556.xx) between July 1, 2009, and June 30, 2013 (study period). Eligible subjects had to have ≥ 2 claims with an IBD diagnosis within 1 year or 1 claim with an IBD diagnosis by a paediatrician or gastroenterologist (using a validated algorithm with a PPV of 93% to 96%) [20]. From this group, individuals with at least 1 prescription claim for a TNFI or immunomodulator were identified.
Chang Y.‐J. (2020) [19]	Impact of rheumatoid arthritis on alopecia: A nationwide population‐based cohort study in Taiwan.	Newly diagnosed RA using the ICD‐9‐CM code 714.0 from 2000 to 2012 AND use of DMARDs ≥ 30 days AND age ≥ 20 years old.
Liu Y. (2024) [21]	Comorbidity burden and health care utilization by substance use disorder patterns among people with HIV in Florida.	The algorithm screens patient's medical records and identifies people with HIV if they had at least one HIV diagnostic code plus at least one of the following: (1) had at least one positive HIV laboratory test, including HIV RNA and antigen/antibody test, (2) had been prescribed ART, or (3) had three or more visits with corresponding HIV diagnostic codes.
Burchell A.N. (2019) [22]	Cause‐specific mortality among HIV‐infected people in Ontario, 1995–2014: A population‐based retrospective cohort study.	The algorithm required 3 physician claims coded for HIV infection [ICD‐9] (codes 042‐044) over a 3‐year period.

Table 3 shows the retrieved medical conditions and medicines organized into seven broad concept categories. *Category* 1 includes 20 genetic and hereditary conditions. *Category* 2 contains seven main infectious diseases and related conditions such as opportunistic fungal infections. *Category* 3 comprises three main diagnostic concepts: hematological malignancies, solid organ malignancies and hospitalization for chemotherapy. *Category* 4 includes four main diagnostic concepts related to solid organ and stem cell transplantation. *Category* 5 groups several medical conditions that were not classified into the other categories, including severe kidney and liver disease, severe malnutrition, severe burns, preterm birth, cryoglobulinemia, asplenia, and hematological neutropenia. *Category* 6 lists immunosuppressants (*n* = 14 medicines or therapeutic groups) and medicines to treat immunosuppressive conditions (*n* = 4 medicines or therapeutic groups). Additionally, *Category* 7 lists 19 autoimmune conditions that may lead to an immunocompromised status only under the evidence of prescription or dispensing of an immunosuppressant.

**TABLE 3:** Clinical conditions and medicinal products retrieved in the literature search and curated by the core study team and clinical experts.

Proposed category	Clinical or drug concept
1. Genetic and hereditary conditions	Immunodeficiency with predominantly antibody defects
	Combined immunodeficiencies
	Common variable immunodeficiency
	Hereditary hypogammaglobulinemia
	Selective immunoglobulin (Ig) M deficiency
	Hyper‐IgM syndromes
	Antibody deficiency with near‐normal immunoglobulins or with hyperimmunoglobulinemia
	Severe combined immunodeficiencies
	Adenosine deaminase (ADA) deficiency
	Nezelof's syndrome
	Purine nucleoside phosphorylase (PNP) deficiency
	Major histocompatibility complex (MHC) class I and II deficiency
	Activated phosphoinositide 3‐kinase delta syndrome
	Wiskott–Aldrich syndrome
	Immunodeficiency with short‐limbed stature
	Immunodeficiency following hereditary defective response to Epstein–Barr virus
	Hyper‐IgE syndromes
	Lymphocyte function antigen‐1 (LFA‐1) defect
	Defects in the complement system
	Congenital asplenia
2. Infectious diseases and related conditions	Human immunodeficiency virus (HIV) infection
	Acquired immune deficiency syndrome (AIDS)
	Immune reconstitution inflammatory syndrome
	Hepatitis C virus (HCV) infection
	Cytomegalovirus (CMV) infection
	Opportunistic mycoses, including infections of the lung and invasive fungal infections (IFI)
	Progressive multifocal leukoencephalopathy (PML)
	*Infections by specific bacteria, virus or fungus in neonates* [23]: – *Sepsis or pneumonia caused by Streptococcus Group B or diagnosis of Escherichia coli* – *Sepsis or meningitis caused by Listeria monocytogenes* – *Encephalitis or disseminated HSV infection* – *Hepatitis, pneumonia, or neurological damage due to CMV* – *Systemic infections due to Candida spp., Cryptoccocus spp., Aspergillus spp*. – *Pneumonia due to Pneumocystis jirovecii*
3. Hematological and solid organ malignancies, and related interventions	Haematologic malignancies, including myelodysplastic syndromes
	Solid organ malignancies
	Hospitalization for chemotherapy
4. Solid organ and hematopoietic stem cell transplantation	Kidney, liver, heart and lung transplant
	Kidney, liver, heart and lung transplant rejection
	Acute and chronic graft‐versus‐host disease
	Hematopoietic stem cell transplantation
5. Other conditions nonclassified elsewhere	End‐stage kidney disease, including dialysis dependency
	Significant liver disease (SLD)
	*Severe malnutrition*
	*Severe burns*
	*Extremely and very preterm birth*
	Cryoglobulinemia
	*Iatrogenic or functional asplenia*
	Hematological neutropenia
6. Immunosuppressants and medicines to treat conditions leading to an immunosuppressive state	Corticosteroids for systemic use (H02)
	Cyclophosphamide (L01AA01)
	Rituximab (L01FA01)
	Azathioprine (L04AX01)
	Methotrexate (L04AX03)
	Selective immunosuppressants (L04AA)
	Tumour necrosis factor alpha (TNF‐α) inhibitors (L04AB)
	Interleukin inhibitors (L04AC)
	Calcineurin inhibitors (L04AD)
	Janus‐associated kinase inhibitors (L04AF)
	Monoclonal antibodies (L04AG)
	*Mammalian target of rapamycin (mTOR) kinase inhibitors* (L04AH)
	*Complement inhibitors* (L04AJ)
	Dihydroorotate dehydrogenase (DHODH) inhibitors (L04AK)
	*Colony stimulating factors* (L03AA)
	Direct acting antivirals (J05A)^a^, with or without combination with cobicistat
	Sulfasalazine (A07EC01)
	Mesalazine (A07EC02)
	Systemic antifungals (J02A)
7. Conditional clinical concepts to be included upon evidence of concomitant use of immunosuppressants	Acquired pure red cell aplasia
	Dermatomyositis
	*Pemphigus vulgaris*
	Pemphigoid
	Cystic fibrosis
	DiGeorge syndrome
	Autoimmune lymphoproliferative syndrome (ALPS)
	IgG4‐related disease
	Hemophagocytic syndromes
	Crohn's disease
	Ulcerative colitis
	Systemic lupus erythematosus
	Systemic sclerosis

	Rheumatoid arthritis
	Psoriatic and enterohepatic arthropathies
	Juvenile arthritis
	Ankylosing spondylitis
	Adult‐onset Still's disease
Systemic arthritis

*Note: Italics*: included upon clinical specialist recommendation.

^a^ Except: nucleosides and nucleotides excl. reverse transcriptase inhibitors (J05AB).

Table 4 presents the proposed modular phenotype algorithm to identify immunocompromised individuals in studies using EHR databases. It includes diagnostic codes, their timing of occurrence, medication exposures, duration and dosage of drug use, diagnosis‐exposure time intervals, diagnostic tests, and use of healthcare resources (e.g., neonatal ICU admission), among others. Table S4 lists the diagnosis codes included in the phenotype algorithm and mapped to the following medical dictionaries: ICD‐10, ICD‐10‐CM, ICD‐10‐DA, ICD‐9, ICD‐9‐CM, ICPC, MEDCODEID, SNOMED international version, and SNOMED Spanish version [13]. Finally, Table S5 exemplifies the implementation of the algorithm according to the ConcePTION Common Data Model (CDM) [31] including the raw logical definitions for each algorithm component, their corresponding temporal relationships and the specific algorithm element to which they apply. Furthermore, the algorithm can be tailored to other existing CDMs.

**TABLE 4:** Phenotype proposal for identifying immunocompromised populations across electronic healthcare data sources.

Exposures^a^	*Prescription or dispensing records of*: (Systemic corticosteroids use (ATC H02) AND (≥ 20 mg for ≥ 2 weeks, up to 3 weeks after stopping OR more than 6 months up to 3 weeks after stopping, independent of the dosage)) [24, 25, 26], OR (Antineoplastic agents (ATC L01) from the start day, up to 6 months after stopping the drug, independent of the dosage), OR (Selective immunosuppressants (ATC L04AA) from 1 month after the start day of the drug, up to 1 month after stopping the drug, independent of the dosage), OR (TNF‐α (ATC L04AB) from 2 weeks after the start day of the drug, up to 2 months after stopping the drug, independent of the dosage) [23, 27], OR (Interleukin inhibitors (ATC L04AC) from 1 month after the start day of the of drug, up to 3 months after stopping the drug, independent of the dosage), OR (Calcineurin inhibitors (ATC L04AD) from 1 week after the start day of the drug, up to 1 week after stopping the drug, independent of the dosage), OR (S1P receptor modulators (ATC L04AE) from 2 weeks after the start day of the drug, up to 2 months after stopping the drug, independent of the dosage), OR (JAK inhibitors (ATC L04AF) from 2 weeks after the start day of the drug, up to 1 week after stopping the drug, independent of the dosage). OR (Monoclonal antibodies (ATC L04AG) from 2 months after the start day of the drug, up to 3 months after stopping the drug, independent of the dosage), OR (mTOR kinase inhibitors (e.g., sirolimus, everolimus) (ATC L04AH) from 2 weeks of after the start day of the drug, up to 2 weeks after stopping the drug, independent of the dosage), OR (Complement inhibitors (e.g., eculizumab) (ATC L04AJ) from 3 days after the start day of the drug, up to 3 months after stopping the drug, independent of the dosage), OR (DHODH inhibitors (e.g., leflunomide) (ATC L04AK), from 1 month after the start day of the drug, up to 5 months after stopping the drug, independent of the dosage), OR (Other immunosuppressants (methotrexate, azathioprine, thalidomide, lenalidomide, pirifenidone, pomalidomide, dardvastrocel, dimethyl fumarate and diroximel fumarate) (ATC L04AX), from 1 month after the start day of the drug, up to 1 week after stopping the treatment, independent of the dosage), OR Colony stimulating factors (e.g., filgrastim, pegfilgrastim) (ATC L03AA), from 5 days before the start day of the drug, up to the last administration day, independent of the dosage, OR (Sulfasalazine (ATC A07EC01) OR mesalazine (ATC A07EC02)) AND any of the diagnoses listed in Category 6, Table 3. OR (Direct acting antivirals, with or without combination with cobicistat (J05A)^a^ AND any of the diagnoses listed in Category 2, Table 3).
Diagnoses (from diagnoses included in Table 3)	(Diagnostic code for genetic and hereditary conditions (Category 1, Table 3) ≤ 5 year prior to the study entry date), **OR** ((Diagnostic code for genetic and hereditary conditions **OR** infectious diseases and related conditions **OR** hematological and solid organ malignancies **OR** solid organ and haematopoietic stem cell transplantation **OR** other conditions not classified elsewhere) **AND** (neutropenia, lymphopenia, leukopenia **OR** any opportunistic infection [22], 1 month apart from the diagnostic code)). **OR** (Diagnostic code for infectious diseases and related conditions (Category 2, Table 3) ≤ 1 year prior to the study entry date). *If HIV disease:* ((*Individuals with at least 1 HIV diagnostic code, **OR** 1 positive HIV test, **OR** HIV antiretroviral therapy prescription of 3‐drug regimens*), *AND* (*at least 1 year of follow‐up, **OR*** ≥ *3 HIV‐related visits*)) ***NOT*** (*2 consecutive viral load measurements of < 400 copies/mL at least 30 days apart within 12 months*), **OR** (Any diagnostic code of sepsis as cause of admission to the hospital NOT present in the previous 3 months) **AND** (exposure to any drug listed in Category 6 Table 3) **OR** (Diagnostic code for hematological and solid organ malignancies, and related interventions): (*if non‐metastatic solid malignancies: ≤ 5 year prior to the study entry date **AND** exposure to any drug listed in Category 6 in Table* *3*), ***OR*** (*if metastatic organ malignancies or haematological malignancies: ≤ 5 year prior to the study entry date*), ***OR***
	(*metastatic organ malignancies or haematological malignancies receiving any of the drugs listed in* Table 4 *±1 month apart from the diagnostic date*), ***OR*** ((*any code of malignant tumour as cause of hospital admission or visit to the hospital **NOT** present in the previous year), **AND** (tumour marker, histologically malignant tumour, and other related tests within 1 month before or after the date of the visit), **OR** (biopsy diagnosis within 1 month before or after the date of the initial visit), **OR** (photographing/imaging within 1 month before or after the date of the initial visit), **OR** (surgery within 3 months after the date of the initial visit), **OR** (prescription or dispensing of antineoplastic drugs (ATC L01) within 3 months after the date of the initial visit), **OR** (radiotherapy within 6 months after the date of the initial visit*)), [28] ***OR*** (*≥ 1 procedure code for bone marrow aspirate, or organ biopsy **AND** prescription or dispensing of antineoplastic drugs (ATC L01) within 3 months after the date of the procedure*) [29] ***OR*** (*Diagnostic code for solid organ and haematopoietic stem cell transplantation* ≤ *5 year prior to the study entry date*), ***OR*** Diagnostic code for other conditions not classified elsewhere: (End‐stage kidney disease, including dialysis dependency, **OR** Haematological neutropenia, **OR** Cryoglobulinemia ≤ 5 year prior to the study entry date), **OR** (SLD [30]: (diagnosis of cirrhosis with ≥ 1 inpatient **OR** ≥ 2 outpatient codes, **OR** decompensated cirrhosis identified by complications like ascites **OR** bleeding oesophageal varices **OR** spontaneous bacterial peritonitis, **OR** HCC identified by cirrhosis diagnosis) **AND** (≥ 1 inpatient **OR** ≥ 2 outpatient codes for HCC, **OR** hepatocellular dysfunction identified by a MELD score ≥ 15, **OR** total serum bilirubin ≥ 3 mg/dL)), **OR** (Diseases related to external conditions including severe malnutrition OR severe burns, ≤ 1 year prior to the study entry date) **OR** (PML diagnosis **AND** an MRI exam performed within 6 months prior the PML diagnosis) [17] **OR** (IFI diagnostic code **OR** record of in‐hospital prescriptions or dispensing of systemic antifungal agents (J02A): fluconazole, itraconazole, posaconazole, voriconazole, amphotericin B, liposomal amphotericin B, caspofungin, micafungin, anidulafungin, and flucytosine) [15]
Diagnoses conditioned to exposures	(Acquired pure red cell aplasia, **OR** dermatomyositis, **OR** pemphigus vulgaris, **OR** pemphigoid, **OR** cystic fibrosis, **OR** DiGeorge syndrome, **OR** ALPS, **OR** IgG4‐related disease, **OR** hemophagocytic syndromes, **OR** Crohn's disease, **OR** ulcerative colitis, **OR** systemic lupus erythematosus, **OR** systemic sclerosis, **OR** rheumatoid arthritis, **OR** psoriatic and enterohepatic arthropathies, **OR** juvenile arthritis, **OR** ankylosing spondylitis, **OR** adult‐onset Still's disease, **OR** systemic arthritis, **OR** Listeria infection) **AND** (Prescription or dispensing records of an immunosuppressant ±1 month apart from diagnosis).
*For pediatric individuals < 1 year of age and neonates, everything stated above*,^*a*^ *AND the following*	
Diagnoses	Diagnostic code for extremely and very preterm birth ≤ 1 year prior to the study entry date. **OR** Diagnostic code for any of the following infections: Bacterial Infections: ((neonate) **AND** ((diagnostic codes of sepsis **OR** pneumonia) **AND** (diagnostic codes of *Streptococcus* Group B infection **OR** *Streptococcus* Group B positive test, **OR** diagnosis of *Escherichia coli* infection **OR** *Escherichia coli* positive test)) **OR** ((diagnostic codes of meningitis **OR** sepsis) **AND** (*Listeria* infection diagnosis **OR** *Listeria monocytogenes* positive test))) [30], **OR** Viral Infections: (neonate) **AND** ((hepatitis **OR** pneumonia **OR** neurologic damage) **AND** (CVM infection diagnosis **OR** CVM positive test)) **OR** (HSV encephalitis **OR** HSV disseminated infection), **OR** Fungal Infections: (neonate) **AND** ((Candida spp., **OR** Aspergillus spp, **OR** Cryptoccocus spp positive test) **OR** (Candida spp., **OR** Cryptoccocus spp, **OR** Aspergillus spp diagnostic code) **OR** (NICU admission)), **OR** Opportunistic Infections: (neonate) **AND** (pneumonia diagnostic code AND (*Pneumocystis jirovecii* positve test **OR** *Pneumocystis jirovecii* infection diagnostic code)).

Abbreviations: ALPS, autoimmune lymphoproliferative syndrome; ATC, Anatomical Therapeutic Chemical classification; CVM, cytomegalovirus; DHODH, dihydroorotate dehydrogenase; HCC, hepatocellular carcinoma; HIV, human immunodeficiency virus; HSV, herpes simplex virus; IFI, in‐hospital fungal infection; JAK, Janus‐associated kinase; MELD, model for end‐stage liver disease score; MRI, magnetic resonance imaging; mTOR, mammalian target of rapamycin; NICU, neonatal intensive care unit; PML, progressive multifocal leukoencephalopathy; S1P, sphingosine‐1‐phosphate; SLD, significant liver disease; TNFα, tumor necrosis factor alpha inhibitors.

^a^ When assessing immunosuppression exposures in children ≤ 12 years of age, lower doses than the ones proposed for adults might be considered as sensitivity analysis.

## 4 Discussion

This study combines the knowledge from a scoping review together with the expertise of clinicians and the advice from the European Medicines Agency to provide a comprehensive overview of the existing operational definitions and to produce a phenotype algorithm to identify immunocompromised individuals in EHR databases. Such a phenotype algorithm is fundamental for properly assessing risk differences in epidemiologic and pharmacoepidemiologic research, especially for vaccine evaluation (e.g., live attenuated vaccines containing weakened forms of the target pathogen may increase the risk of causing the disease in immunocompromised individuals as compared with those with healthy immune systems) [32, 33]. The proposed phenotype algorithm has the potential to allow for a broad mapping of related diagnostic codes, tests, and medicinal products. In this sense, depending on the population of interest, the available type data sources, and the research question, it can be used by pooling all algorithm blocks together or by selecting individual elements (e.g., by omitting algorithm components related to treatment duration, if unavailable), making the algorithm flexible enough to be used in different settings (intensive care, hospital, or primary care) and to address different research questions. Additionally, the algorithm's broadness and modularity support the identification of immunocompromised populations, as it can be tailored depending on data characteristics (by capturing nuances such as corticosteroid dosage, exposure duration, or test results). Of note, the exclusion of components may increase the risk of misclassification, even if the algorithm remains suitable for generating real‐world evidence.

The dynamic nature of acquired immunocompromised status challenges the task of defining its exact duration. For instance, immunodeficiency may be temporary in several secondary immunodeficiencies, resolved once treatment concludes or when the disease enters remission. The proposed algorithm considers this variability by stating the time‐period(s) used to define the immunocompromised status for each category of conditions. Moreover, certain medications can serve as indirect indicators of an immunocompromised state, for example, systemic antifungal agents or antiviral drugs, if combined with relevant clinical conditions. Although the dose information was not considered for all medicines included in the phenotype algorithm, the dosage of systemic corticosteroids was included due to the high prevalence of use of systemic corticosteroids among immunocompromised populations. Corticosteroids can have immunosuppressive effects with dose and duration being crucial factors. Doses of prednisone or equivalent ≥ 20 mg/day for ≥ 2 weeks or > 40 mg/day for > 1 week are considered immunosuppressive [24, 25, 26]. These doses can significantly reduce B‐ and T‐lymphocyte subpopulations and affect immune function, potentially lasting for weeks or months after discontinuation. As the drug's dosage information is not always available in data sources, the phenotype algorithm combines the possibility of capturing information on corticosteroids' dosage or time windows around the prescription or dispensing record, or on the exposure time only. Overall, in this study, immunocompromised status was defined to reflect active cases around the index date, rather than a lifetime history of immunocompromising conditions. Accordingly, this algorithm is primarily suited for analyses focusing on active immunocompromised status.

The proposed algorithm also covers the specific features of paediatric sub‐populations. As children and newborns might be subject to specific health conditions since the immune system reaches adult maturity after 12 years of age [34, 35, 36]. Immunocompromised newborns are susceptible to a wide range of pathogens, including common and opportunistic organisms [20, 36, 37]. Hence, we proposed a neonatal variation of the algorithm, including specific infections, neonatal outcomes (such as preterm birth), and adjustments on the dose and time‐window thresholds.

## 5 Limitations

The search strategy of this scoping review (Table S1) has some limitations as the search terms may not be found explicitly in publication titles or abstracts but in the methods and Supporting Information files instead. Moreover, novel concepts not yet indexed in literature databases might not have been captured. For instance, the increased understanding of human immunology and genetics linked to improved laboratory techniques, mainly genomic tools, has produced an important expansion of new PID phenotypes in recent years [38]. Although the proposed algorithm captures an important spectrum of PIDs, it may miss very rare or newly defined PIDs [39]. Finally, due to the rarity of several of these diseases, the newest therapeutic alternatives, such as gene therapy, enzyme replacement therapy, or therapies without an assigned ATC to treat severe combined immunodeficiencies, have not been identified in our literature search. These therapies are rarely studied using observational databases due to the limited availability of recorded data, especially when they have recently been commercialized [40].

Regarding limitations of the proposed phenotype algorithm, the transient nature of some immunocompromised conditions represents a main challenge. Immunocompromising situations can be transient, resolving as treatment concludes, or diseases enter remission. Although we sought to account for this variability by including time‐sensitive criteria, such as specifying time windows for drug exposure or diagnostic testing, capture of these transitions may be limited by the availability of data in EHR data sources, and the duration of exposure might be misclassified. We included a proposal to deal with this challenge by adding dose and duration recommendations for systemic corticosteroids, a therapeutic group widely used in this population. However, for the remaining medicines, we have added the immunosuppressive duration of exposure but not the dosage information due to the complexity of giving accurate recommendations. Another important limitation lies in the fact that the performance of the phenotype algorithm still needs to be tested across diverse healthcare settings, systems and data sources [12]. For example, administrative and claims‐based databases offer broad capture of healthcare encounters across settings, whereas EHR‐based data sources provide richer clinical detail. Thus, future work could examine how data structure, coding practices, and care settings of different data source types (e.g., administrative/claims versus EHR‐based datasets) influence algorithm validity and transportability. Finally, the algorithm may require refinement to account for other subpopulations, that is, pregnant persons and children aged 1–12 years. Moreover, while studies involving older adults were considered, individuals aged ≥ 65 years may not necessarily be classified as immunocompromised per se [1, 12]. However, they warrant special consideration [12, 41, 42, 43, 44] due to the unique characteristics of their immune systems, particularly the onset of immunosenescence [1, 2, 45]. Future studies could also explore additional situational and biosocial factors, such as bedridden states, long‐term immobilization, chronic illnesses with extended progression, high frailty levels, and those in marginalized social conditions with limited access to food and healthcare.

## 6 Conclusions

A proper phenotype algorithm to identify immunocompromised individuals is crucial in epidemiologic and pharmacoepidemiologic research. Supported by findings from a targeted literature search, we developed a phenotype algorithm to identify immunocompromised individuals when conducting research using EHR data sources. A variety of clinical conditions, medicines, and laboratory tests were joined using sequential logic. Considering the diversity of EHR data sources, the phenotype algorithm was built using a modular strategy by putting algorithm blocks together, enhancing adaptability across diverse data source types and research questions. A primary challenge when attempting to identify individuals with immunocompromised status is the dynamic nature of secondary immunodeficiencies since several conditions may be transient in time. The phenotype algorithm presented here partially addresses this challenge by considering the time‐period(s) most likely to define an individual's immunocompromised status. Future research should formally test the proposed algorithm.

## Author Contributions

All authors contributed to the study conception and design. J.R.‐A. managed the software and questionnaire for data extraction. Literature search and data extraction were performed by J.R.‐A., N.L., F.R., I.P., C.E.D., Ma.S., and S.M. E.B. gave support to the literature search and scientific discussion. L.S.S., L.C., E.M.‐M., E.B., and M.L.‐L. gave relevant clinical expert advice. A.P. and C.C. provided relevant regulatory and scientific input. The first draft of the manuscript was written by J.R.‐A., C.E.D., F.R., and I.P., and all authors commented on previous versions of the manuscript. All authors reviewed and approved the final manuscript.

## Funding

This project received support from the European Medicines Agency under the Framework service contract no. EMA/2020/46/TDA/L5.06.

## Ethics Statement

The authors have nothing to report.

## Consent

This research is based on secondary use of anonymized healthcare data. Informed consent to participate is therefore not required. The study was conducted in compliance with the ENCePP Code of Conduct.

## Conflicts of Interest

The research leading to these results was conducted as part of the activities of the EU PE&PV (Pharmacoepidemiology and Pharmacovigilance) Research Network (led by Utrecht University) in collaboration with the Vaccine Monitoring Collaboration for Europe network (VAC4EU). The University Medical Center Utrecht (UMCU) coordinated the scientific work for this project. The project has received support from the European Medicines Agency under the Framework service contract no. EMA/2020/46/TDA/L5.06 and was conducted following the ENCePP code of conduct. This manuscript expresses the opinion of the authors and may not be understood or quoted as being made on behalf of or reflecting the position of the European Medicines Agency or one of its committees or working parties. This study has been registered in the HMA‐EMA Catalogue of Real‐World Data under the EU PAS number: EUPAS1000000288.

## Supporting information


**Table S1:** Search string.
**Table S2:** Articles selected for data extraction.
**Table S3:** Frequency of citation of diagnoses and drugs or therapeutic groups.
**Table S4:** List of diagnostic and procedure codes included in the immunocompromise phenotype algorithm, mapped across different coding systems (CSV file).
**Table S5:** Immunocompromise phenotype algorithm logic according to the ConcePTION CDM (Excel file).
**Table S6:** List of the SAFETY‐VAC study consortium members.

**Data S1:** pds70362‐sup‐0002‐Supinfo1.xlsx.

**Data S2:** pds70362‐sup‐0003‐Supinfo2.csv.
