# Supplementary material for: Immunocompromised Status Definition in Observational Studies Using Electronic Health Records: A Scoping Review and a Proposal for a Phenotype Identification Algorithm
Source: Pharmacoepidemiol Drug Saf. 2026 Mar 27;35(4):e70362. doi: 10.1002/pds.70362 (PMC13031886; doi:10.1002/pds.70362)
Supplement: Supplementary file 1 — Table S1: Search string. Table S2: Articles selected for data extraction. Table S3: Frequency of citation of diagnoses and drugs or therapeutic groups. Table S4: List of diagnostic and procedure codes included in the immunocompromise phenotype algorithm, mapped across different coding systems (CSV file). Table S5: Immunocompromise phenotype algorithm logic according to the ConcePTION CDM (Excel file). Table S6: List of the SAFETY‐VAC study consortium members. [file PDS-35-e70362-s001.pdf]

Supplementary material

Immunocompromised Status Definition in Observational Studies using Electronic Health Records: A Scoping Review and a Proposal for a Phenotype Identification Algorithm.

Table of Contents

Supplementary Table 1. Search string 2

Supplementary Table 2. Articles selected for data extraction 4

Supplementary Table 3. Frequency of citation of diagnoses and drugs or therapeutic groups 10

Supplementary Table 4 (CSV file). List of diagnostic and procedure codes included in the immunocompromise phenotype algorithm, mapped across different coding systems 13

Supplementary Table 5 (Excel file). Immunocompromise Phenotype Algorithm Logic according to the ConcePTION CDM 14

Supplementary Table 6. List of the SAFETY-VAC study consortium members 15

Supplementary Table 1. *Search string*

#1	diagnostic codes[All Fields] OR "ATC"[All Fields] OR "coding system"[All Fields] OR "standard code"[All Fields] OR "medical vocabulary"[All Fields] OR "medical dictionary"[All Fields] OR "International Classification of Diseases"[All Fields] OR (("anatomic"[All Fields] OR "anatomical"[All Fields] OR "anatomically"[All Fields]) AND ("therapeutical"[All Fields] OR "therapeutically"[All Fields] OR "therapeuticals"[All Fields] OR "therapeutics"[MeSH Terms] OR "therapeutics"[All Fields] OR "therapeutic"[All Fields]) AND ("chemical"[All Fields] OR "chemical s"[All Fields] OR "chemically"[All Fields] OR "chemicals"[All Fields]) AND "ATC"[All Fields] AND ("clinical coding"[MeSH Terms] OR ("clinical"[All Fields] AND "coding"[All Fields]) OR "clinical coding"[All Fields] OR "code"[All Fields]) AND ("system"[All Fields] OR "system s"[All Fields] OR "systems"[All Fields])) OR "atc code*"[All Fields] OR "anatomical therapeutic chemical code"[All Fields] OR "medicin* code"[All Fields] OR "drug code"[All Fields] OR "systematized nomenclature of medicine"[MeSH Terms] OR "vocabulary, controlled"[MeSH Terms] OR "International Classification of Diseases"[MeSH Terms] OR "unified medical language system"[MeSH Terms] OR "logical observation identifiers names and codes"[MeSH Terms] OR "standardized nursing terminology"[MeSH Terms] OR "diagnostic and statistical manual of mental disorders"[MeSH Terms] OR "current procedural terminology"[MeSH Terms]	73,540
#2	"Immunocompromised Host"[Mesh] OR "Immunosup*"[Title/Abstract] OR "Immunosuppressive Agents"[Mesh] OR "L04"[Title/Abstract] OR "Immunosuppression Therapy"[Mesh] OR "Immunocompr*"[Title/Abstract] OR "Immunosup*"[All fields] OR "corticoster*"[Title/Abstract] OR "antineoplas*"[Title/Abstract] OR "chemother"[Title/Abstract] OR "antiretrovir*"[Title/Abstract] OR "AIDS"[Title/Abstract] OR "HIV"[Title/Abstract] OR "transplant*"[Title/Abstract] OR "trasplant*"[Title/Abstract] OR "immunodef*"[Title/Abstract]	1,472,526
#3	"epidemiolog*"[MeSH Terms] OR "Pharmacoepidemiology"[MeSH Terms] OR "retrospective"[Title/Abstract] OR "cohort*"[Title/Abstract] OR "longitudinal studies"[Title/Abstract] OR "cross sectional"[Title/Abstract] OR "cross-sectional"[Title/Abstract] OR "pharmacoepidemiologic"[Title/Abstract] OR "pharmacoepidemiological"[Title/Abstract] OR "case-control"[Title/Abstract] OR "case control"[Title/Abstract] OR "case-crossover"[Title/Abstract] OR "case crossover"[Title/Abstract] OR "case time-control"[Title/Abstract] OR "case-time-control"[Title/Abstract] OR "case-time control"[Title/Abstract] OR "self-controlled case series"[Title/Abstract] OR "self controlled case series"[Title/Abstract] OR "self-controlled risk interval"[Title/Abstract] OR "SCRI"[Title/Abstract] OR "SCCS"[Title/Abstract] OR "times series"[Title/Abstract] OR "new user active comparator design"[Title/Abstract] OR "new-user active comparator design"[Title/Abstract]	2,210,208
#4	"database*"[Title/Abstract] OR "Databases"[Title/Abstract] OR "data bases"[Title/Abstract] OR "computerized data"[Title/Abstract] OR "administrative claims"[Title/Abstract] OR "administrative data"[Title/Abstract] OR "claims data*"[Title/Abstract] OR "healthcare records"[Title/Abstract] OR "health records"[Title/Abstract] OR "electronic health records"[Title/Abstract] OR "data bases"[Title/Abstract] OR "databases pharmaceutical"[Title/Abstract] OR "electronic healthcare database"[Title/Abstract] OR "healthcare databases"[Title/Abstract] OR "drug utilisation database"[Title/Abstract] OR "drug use database"[Title/Abstract] OR "Databases, Factual"[Mesh] OR "multiple databases"[Title/Abstract]	1,070,552

	OR "databases"[Title/Abstract] OR "multi-database"[Title/Abstract] OR "Multi-Database"[Title/Abstract] OR "multidatabase"[Title/Abstract] OR "multi-database"[Title/Abstract] OR "multi-source"[Title/Abstract] OR "multi cent*"[Title/Abstract] OR "multinational"[Title/Abstract] OR "multi-cohort"[Title/Abstract] OR "multi-site"[Title/Abstract] OR "multiple sites"[Title/Abstract] OR "distributed data*"[Title/Abstract] OR "distributed network"[Title/Abstract] OR "distributed data network"[Title/Abstract] OR "database network"[Title/Abstract] OR "data network"[Title/Abstract] OR "research network"[Title/Abstract] OR "safety network"[Title/Abstract] OR "MDBS"[Title/Abstract] OR "MDPES"[Title/Abstract] OR "cohorts"[Title/Abstract]	
#5	"clinical trials"[Title/Abstract] OR "pre-clinical"[Title/Abstract] OR "in vitro"[Title/Abstract] OR "preclinical"[Title/Abstract] OR "Phase I"[Title/Abstract] OR "Phase II"[Title/Abstract] OR "Phase III"[Title/Abstract] OR "Phase 1"[Title/Abstract] OR "Phase 2"[Title/Abstract] OR "Phase 3"[Title/Abstract] OR "in-vitro"[Title/Abstract] OR "in silico"[Title/Abstract] OR "double-blind"[Title/Abstract] OR "placebo-controlled"[Title/Abstract] OR "single centre"[Title] OR "single center"[Title] OR "Single-Centre"[Title] OR "Single-Center"[Title] OR "pilot trial"[Title/Abstract] OR "randomized controlled trial"[Title/Abstract] OR "randomized controlled trials"[Title/Abstract] OR "randomised controlled trial"[Title/Abstract] OR "randomised controlled trials"[Title/Abstract] OR "clinical trial"[Title/Abstract] OR "controlled clinical trial"[Title/Abstract] OR "controlled trial"[Title/Abstract] OR "randomized clinical trial"[Title/Abstract] OR "randomised clinical trial"[Title/Abstract] OR "animal"[Title/Abstract] OR "RCT"[Title/Abstract] OR "experimental"[Title/Abstract] OR "cell"[Title/Abstract] OR "celular"[Title/Abstract] OR "clinical practice guideline"[Title/Abstract] OR "case series"[Title/Abstract] OR "systematic review"[Title/Abstract] OR "systemic review"[Title/Abstract] OR "literature review"[Title/Abstract] OR "narrative review"[Title/Abstract] OR "scoping review"[Title/Abstract] OR "documentary search"[Title/Abstract] OR "Editorial"[Publication Type] OR "Review"[Publication Type] OR "Practice Guideline"[Publication Type] OR "Published Erratum"[Publication Type] OR "surveys and questionnaires"[MeSH Terms] OR "data collection"[MeSH Terms] OR "protocol"[Title] OR "guideline"[Title] OR "case report"[Title/Abstract] OR "case reports"[Title/Abstract] OR "Case Reports"[Publication Type] OR "Validation Study"[Publication Type] OR "Consensus Development Conference"[Publication Type] OR "Clinical Conference"[Publication Type] OR "Congress" [Publication Type] OR "Congress"[Publication Type] OR "Guideline"[Publication Type] OR "News"[Publication Type] OR "Letter"[Publication Type] OR "Review"[Publication Type] OR "randomized controlled trial"[Publication Type] OR "case reports"[Publication Type] OR "editorial"[Publication Type] OR "review"[Publication Type] OR "systematic review"[Publication Type] OR "primary collection"[All fields] OR "omic*"[Title/Abstract] OR "econom*"[Title/Abstract] OR "pharmacoeconom*"[Title/Abstract] OR "Economics, Pharmaceutical"[Mesh] OR OR "Health Care Economics and Organizations"[Mesh]	17,539,666
	**(#1 AND #2 AND #3 AND #4) NOT #5**	137

Supplementary Table 2. *Articles selected for data extraction*

Author	Year	DOI	Main study topic	Study country/ies	Num. of databases	Type of database	Subjects included in the study	Population being studied	Item to define immunocompromised population		Indication of whether the codes were previously validated or validated in the current study
									Item	Vocabularies	
Kulaylat	2017	10.1001/jamasurg.2017.1538	Safety	US	1	Administrative/Claims	2476	Adults, Elders	Diagnostic	ICD-9-CM	Not specified
									Medicinal product	Vocabulary not reported	-
James	2017	10.1002/acr.23142	Drug utilization	US	1	Administrative/Claims	393	Paediatrics	Medicinal product	ATC	-
Farraj	2022	10.1002/jgh3.12841	Drug utilization	US	1	Administrative inhospital	283970	Any population or no specific exclusions reported	Diagnostic	ICD-10	Not specified
Liu	2024	10.1007/s10461-024-04325-y	Epidemiology, descriptive	US	1	EHR linked to claims	42271	Adults	Algorithm	-	Validated
Singh	2022	10.1007/s10620-021-07073-4	Safety	US	1	Administrative/Claims	5566	Adults	Diagnostic	ICD-9, ICD-10	Not specified
									Medicinal product	ATC	-
Perrone	2020	10.1007/s40744-020-00218-3	Drug utilization	Italy	4	All Administrative	41290	Adults, Elders	Diagnostic	ICD-9-CM, Exemption codes	Not specified
									Medicinal product	ATC	-
Cavanaugh	2015	10.1016/j.arth.2015.09.003	Epidemiology, descriptive	US	1	Administrative inhospital	1016686	Any population or no specific exclusions reported	Diagnostic	ICD-9-CM	Not specified
Ahlquist	2023	10.1016/j.arth.2023.05.028	Safety	US	1	Administrative inhospital	13611	Adults	Diagnostic	ICD-9-CM, ICD-10-CM	Not specified
Goldberg	2016	10.1016/j.cgh.2016.06.019	Epidemiology, descriptive and association	US	2	All Administrative/Claims	84530	Adults	Algorithm	-	Validated
Cotter	2017	10.1016/j.cgh.2016.11.037	Safety	US	3	EHR and EMR	937	Adults, Paediatrics	Diagnostic	Vocabulary not reported	Validated
									Medicinal product		-

									Clinical definition		-
Abdelhay	2023	10.1016/j.eclinm.2023.102143	Epidemiology, descriptive	US	1	Administrative inhospital	16136	Adults	Diagnostic	ICD-9-CM, ICD-10-CM	Not specified
Liao	2018	10.1016/j.jcma.2018.04.003	Epidemiology, descriptive	Taiwan	1	Administrative/Claims	328	Adults, Elders	Diagnostic	ICD-9-CM	Not specified
									Medicinal product	ATC	-
Cho	2020	10.1016/j.jdin.2020.05.002	Epidemiology, descriptive	Taiwan	1	Administrative/Claims	12780	Any population or no specific exclusions reported	Diagnostic	ICD-9-CM	Not specified
									Medicinal product	ATC	-
Kavcic	2013	10.1016/j.jpeds.2012.11.038	Epidemiology, descriptive	US	1	EHR/Administrative	2875	Paediatrics	Diagnostic	ICD-9-CM	Not validated
									Medicinal product	ATC	-
Bala	2016	10.1016/j.jse.2016.02.033	Epidemiology, descriptive	US	1	Administrative/Claims	91555	Adults, Elders	Diagnostic	ICD-9	Not specified
Chiou	2023	10.1016/j.jseint.2023.04.008	Epidemiology, descriptive	US	1	Administrative/Claims	1191	Any population or no specific exclusions reported	Clinical definition	-	-
Tran	2022	10.1016/j.jvs.2021.09.034	Safety	US	1	Administrative inhospital	224912	Adults, Elders	Diagnostic	ICD-9-CM, ICD-10-CM	Not specified
									Clinical definition	-	-
Kolbrink	2022	10.1016/j.lanepe.2022.100400	Epidemiology, descriptive	Germany	1	Administrative inpatient	Not reported	Any population or no specific exclusions reported	Diagnostic	ICD-10	Not specified
									Clinical definition	-	-
Tanenbaum	2018	10.1016/j.spinee.2017.11.007	Epidemiology, descriptive	US	1	Administrative inhospital	514572	Adults, Elders	Diagnostic	ICD-9-CM	Not specified
									Clinical definition	-	-
Santos	2015	10.1016/j.transproceed.2015.04.087	Coding system validation	US	1	EHR	393	Adults	Diagnostic	ICD-9-CM	Validated
									Clinical definition	-	-
									Population specifications:		-

									subjects with confirmed kidney transplantation and readmission to the hospital		
Joly	2022	10.1093/brain/awac237	Algorithm validation and Epidemiology, descriptive	France	1	Administrative/EHR	584	Any population or no specific exclusions reported	Diagnostic	ICD-10	Validated
									Population specifications: presence of a predisposing immunosuppressive condition ICD-10 code, either in public or private hospital discharge databases	-	-
									Algorithm		Validated
George	2020	10.1093/cid/ciaa284	Drug Utilization	US	1	Registry	8315	Adults	Diagnostic	ICD-9, ICD-10, CSS code	Validated
									Medicinal product	ATC	-
Gregory	2019	10.1093/ecco-jcc/jjy148	Safety	US	1	Administrative/Claims	16005	Adults, Elders, Pregnant women	Diagnostic	ICD-9-CM, ICD-9-CMPCS, CPT-4	Not specified
									Medicinal product	ATC	-
Davy-Mendez	2021	10.1093/infdis/jiaa786	Safety	Canada	1	Registry	6997	Any population or no specific exclusions reported	Diagnostic	ICD-9, ICD-9-CM	Not specified
									Medicinal product	ATC	-
									Clinical definition	-	-
Katrak	2016	10.1093/ofid/ofw173	Epidemiology, descriptive	US	1	Administrative/Claims	377021	Any population or no specific exclusions reported	Diagnostic	ICD-9, LOINC	Validated
									Clinical definition	-	-
Grau	2018	10.1097/BOT.0000000000001286	Safety	US	1	EHR inhospital	4717536	Any population or no specific exclusions reported	Diagnostic	ICD-9-CM	Not specified
									Population specifications: people admitted due to femoral fractures	-	-
King	2012	10.1097/BRS.0b013e318228f32d	Epidemiology, descriptive	US	1	Registry/Administrative	119077	Adults, Elders	Diagnostic	ICD-9, ICD-9-CM	Not specified
									Medicinal product	Vocabulary not reported	-
Moffett	2014		Drug Utilization	US	1		466	Paediatrics	Diagnostic	ICD-9	Validated

		10.1097/MAT.0000000000000037				Administrative			Medicinal product	Vocabulary not reported	-
Asfari	2020	10.1097/MEG.0000000000001681	Epidemiology, association	US	1	Administrative inhospital	30712524	Adults, Elders	Diagnostic	ICD-9	Not specified
Chakraborty	2020	10.1097/QAD.0000000000002384	Drug Utilization	US	1	Registry/Administrative	27216	Adults	Medicinal product	Dictionary not reported	-
									Clinical definition	-	-
Santos	2016	10.1097/TXD.0000000000000573	Epidemiology, association	US	1	Administrative/Claims ihospital	7912	Adults, Elders	Diagnostic	ICD-9-CM	Validated
									Other	-	-
Chen	2018	10.1111/1756-185X.13246	Epidemiology, association	Taiwan	1	Administrative/Claims	71650	Adults	Diagnostic	ICD-9-CM	Not specified
Chládek	2013	10.1111/j.1468-3083.2012.04643.x	Safety	Czech Republic	1	EMR	49	Adults	Diagnostic	Vocabulary not reported	Not specified
									Medicinal product	ATC	-
Alqahtani	2018	10.1111/pace.13498	Epidemiology, descriptive	US	1	Administrative inhospital	1147760	Adults, Elders	Diagnostic	ICD-9-CM	Not specified
									Clinical definition	-	-
Tsao	2019	10.1136/bmjopen-2018-023714	Safety	Canada	4	Administrative, Claims, EHR and Registry	6218	Pregnant women	Diagnostic	ICD-9, ICD-10	Not specified
									Medicinal product	ATC	-
									Clinical definition	-	-
Ng	2022	10.1136/rmdopen-2022-002343	Epidemiology, association	Taiwan	1	Administrative/Claims	319	Adults	Diagnostic	ICD-9-CM	Not specified
									Medicinal product	ATC	-
Massicotte-Azarniouch	2020	10.1177/2054358120977390	Diagnostic codes validation	Canada	1	Registry	1258	Adults	Diagnostic	ICD-10	Validated
Langley	2010	10.1186/1471-2334-10-219	Effectiveness	Canada	2	All administrative/Claims	879	Any population or no specific exclusions reported	Diagnostic	ICD-9-CM	Validated
									Clinical definition	-	-
Zilberberg	2014	10.1186/s13054-014-0590-1	Epidemiology, descriptive	US	1	EHR	10839	Adults, Elders	Diagnostic	ICD-9-CM	Not specified
									Medicinal product	ATC	-

									Clinical definition	-	-
Triant	2007	10.1210/jc.2006-2190	Epidemiology, association	US	1	EHR/registry	2093029	Adults	Diagnostic	ICD-9-CM	Not specified
Chow	2014	10.1212/WNL.0000000000000958	Epidemiology, association	US	1	Registry	39519	Adults, Elders	Diagnostic	ICD-9-CM	Validated
Mangia	2011	10.1371/journal.pone.0014817	Epidemiology, descriptive	Brazil	1	Administrative EHR	55370457	Paediatrics	Diagnostic	ICD-9, ICD-10	Validated
									Medicinal product	Vocabulary not reported	-
									Clinical definition	-	-
Cammarota	2018	10.2147/CEOR.S162625	Epidemiology, descriptive	Italy	1	EHR	1026	Adults	Diagnostic	ICD-9-CM	Not specified
									Clinical definition	-	-
Chang	2020	10.3389/fmed.2020.00150	Epidemiology, association	Taiwan	1	Administrative EHR	5810	Adults, Elders	Diagnostic	ICD-9-CM	Validated
									Medicinal product	ATC	-
									Clinical definition	-	-
									Algorithm		-
Rider	2019	10.3389/fped.2019.00070	Safety	US	1	Administrative/Claims	185892	Adults, Paediatrics	Diagnostic	ICD-9, ICD-10	Not specified
									Clinical definition	-	-
Edigin	2020	10.7759/cureus.9873	Epidemiology, descriptive	US	1	EMR	112	Adults	Diagnostic	ICD-9	Validated
Burchell	2019	10.9778/cmajo.20180159	Epidemiology, descriptive	Canada	6	Five administrative/claims and one EHR	23043	Adults, Paediatrics, Elders	Algorithm	-	Validated
Kroner	2019	10.1016/j.mayocpiqo.2019.03.006	Epidemiology, descriptive	US	1	Administrative inhospital	433805	Adults	Diagnostic	ICD-9-CM	Validated
									Clinical definition	-	-
Wright	2022	10.1016/S2213-2600(22)00042-X	Effectiveness	US	1	EMR (from 42 hospitals)	9667	Adults	Diagnostic	ICD-10-CM	Not specified
Lenert	2020	10.1093/rheumatology/kez622	Epidemiology, descriptive	US	1	Administrative/Claims	636	Adults	Diagnostic	ICD-9-CM, ICD-10-CM	Not validated
Chin-Fang	2021	10.1177%2F1759720X211058502	Epidemiology, association	Taiwan	1	Administrative/Claims	269951	Any population or no specific	Diagnostic	ICD-9-CM	Validated
									Medicinal product	ATC	-

								exclusions reported	Algorithm	-	-
Orieux	2024	10.1186/s13054-023-04774-2	Epidemiology, descriptive	France	1	EMR (from 37 hospitals)	222	Adults	Population specifications: chronic use of immunosuppressive drugs, cancer or hematologic malignancy, and chronic kidney disease stage 3	-	-
Tseng	2019	Doi not available. PMID: 31074727	Safety	Taiwan	1	Administrative/Claims	19603	Adults, Elders	Diagnostic	ICD-9-CM	Not specified
									Medicinal product	ATC	-
Lee	2018	10.1093/ibd/izx080	Safety	US	1	Administrative/Claims	10838	Adults, Paediatrics	Diagnostic	-	-
									Medicinal product	-	-
									Algorithm	-	Validated
Moein	2023	10.6002/ect.2023.0137	Safety	US	1	EHR	96	Adults	Diagnostic	Not reported	Not specified
									Medicinal product		-
Dregan	2015	10.3233/jad-150171	Epidemiology, association	United Kingdom	1	EHR	466976	Adults, Elders	Diagnostic	Vocabulary not reported	Not specified
									Medicinal product		-

ATC: Anatomical Therapeutic Chemical (code); CM: Clinical Modification; DOI: Digital Object Identifier; EHR: Electronic Healthcare Records; EMR: Electronic Medical Records; ICD: International Classification of Diseases; US: United States

Supplementary Table 3. *Frequency of citation of diagnoses and drugs or therapeutic groups*

Num.	Clinical and drug entities	Num. of studies citing the entity (%)	Articles included in our review
1	HIV infection or AIDS	10 (17.9%)	10.1016/j.jse.2016.02.033, 10.1016/j.jvs.2021.09.034, 10.1093/ofid/ofw173, 10.1212/WNL.0000000000000958, 10.9778/cmajo.20180159, 10.1097/BOT.0000000000001286, 10.1210/jc.2006-2190, 10.2147/CEOR.S162625, 10.1016/j.lanepe.2022.100400, 10.1007/s10461-024-04325-y
2	Kidney, liver, heart and lung transplant status or rejection	8 (14.3%)	10.1016/j.arth.2023.05.028, 10.1016/j.lanepe.2022.100400, 10.1177/2054358120977390, 10.1016/j.arth.2015.09.003, 10.1016/j.jpeds.2012.11.038, /10.1016/j.mayocpiqo.2019.03.006, 10.1111/pace.13498, 10.1016/j.transproceed.2015.04.087
3	Methotrexate (L04AX03)	7 (12.5%)	10.1016/j.jdin.2020.05.002, 10.1093/ecco-jcc/jjy148, 10.1136/rmdopen-2022-002343, 10.3389/fmed.2020.00150, 10.1007/s40744-020-00218-3, 10.3233/jad-150171, 10.1093/rheumatology/kez622,
4	Corticosteroids for systemic use (H02)	6 (10.7%)	10.1007/s40744-020-00218-3, 10.3233/jad-150171, 10.3389/fmed.2020.00150, 10.1093/rheumatology/kez622, /10.1186/s13054-023-04774-2, 10.1002/jgh3.12841
5	Selective immunosuppressants (L04AA)	6 (10.7%)	10.1093/ecco-jcc/jjy148, 10.1136/rmdopen-2022-002343, 10.6002/ect.2023.0137, 10.1007/s10620-021-07073-4, 10.1007/s40744-020-00218-3, 10.1016/j.jcma.2018.04.003
6	TNF-α inhibitors (L04AB)	6 (10.7%)	10.1093/ibd/izx080, 10.1136/bmjopen-2018-023714, 10.1136/rmdopen-2022-002343, 10.1007/s10620-021-07073-4, 10.1007/s40744-020-00218-3, 10.1093/ecco-jcc/jjy148,
7	Calcineurin inhibitors (L04AD)	6 (10.7%)	10.1007/s40744-020-00218-3, 10.1016/j.jcma.2018.04.003, 10.1136/rmdopen-2022-002343, 10.3233/jad-150171, 10.6002/ect.2023.0137 /10.1093/rheumatology/kez622
8	End-stage kidney disease, including dialysis dependency	5 (8.9%)	10.1016/j.arth.2015.09.003, 10.1016/j.arth.2023.05.028, /10.1186/s13054-023-04774-2, 10.1016/j.lanepe.2022.100400, 10.1111/pace.13498
9	Azathioprine (L04AX01)	4 (7.1%)	10.1093/ecco-jcc/jjy148, 10.1093/ibd/izx080, 10.3233/jad-150171, /10.1093/rheumatology/kez622
10	Rituximab (L01FA01)	3 (5.4%)	10.1016/j.jpeds.2012.11.038, 10.1136/rmdopen-2022-002343, 10.1007/s40744-020-00218-3
11	Solid organ malignancies	3 (5.4%)	10.1016/j.lanepe.2022.100400, 10.1016/j.jpeds.2012.11.038, /10.1186/s13054-023-04774-2
12	Haematologic malignancies, including myelodysplastic syndromes	3 (5.4%)	10.1016/j.lanepe.2022.100400, 10.1016/j.jpeds.2012.11.038, /10.1186/s13054-023-04774-2
13	Hepatitis C virus (HCV) infection	3 (5.4%)	10.1093/ofid/ofw173, 10.1097/BOT.0000000000001286, 10.2147/CEOR.S162625
14	Combined immunodeficiencies	2 (3.6%)	10.1016/S2213-2600(22)00042-X, 10.1016/j.lanepe.2022.100400

15	Cytomegalovirus (CMV) infection	2 (3.6%)	10.1016/j.transproceed.2015.04.087, 10.1016/j.jvs.2021.09.034
16	Opportunistic mycoses, including infections of the lung and invasive fungal infections (IFI)	2 (3.6%)	10.1177%2F1759720X211058502, 10.1016/j.jvs.2021.09.034
17	Interleukin inhibitors (L04AC)	2 (3.6%)	10.1136/rmdopen-2022-002343, 10.1007/s40744-020-00218-3
18	Immunodeficiency with predominantly antibody defects	1 (1.8%)	10.1016/S2213-2600(22)00042-X
19	Hereditary hypogammaglobulinemia	1 (1.8%)	10.1016/S2213-2600(22)00042-X
20	Common variable immunodeficiency	1 (1.8%)	10.1016/S2213-2600(22)00042-X
21	Selective immunoglobulin (Ig) M deficiency	1 (1.8%)	10.1016/S2213-2600(22)00042-X
22	Hyper-IgM syndromes	1 (1.8%)	10.1016/S2213-2600(22)00042-X
23	Antibody deficiency with near-normal immunoglobulins or with hyperimmunoglobulinemia	1 (1.8%)	10.1016/S2213-2600(22)00042-X
24	Severe combined immunodeficiencies	1 (1.8%)	10.1016/S2213-2600(22)00042-X
25	ADA deficiency	1 (1.8%)	10.1016/S2213-2600(22)00042-X
26	Nezelofs syndrome	1 (1.8%)	10.1016/S2213-2600(22)00042-X
27	PNP deficiency	1 (1.8%)	10.1016/S2213-2600(22)00042-X
28	MHC class I & II deficiency	1 (1.8%)	10.1016/S2213-2600(22)00042-X
29	Activated phosphoinositide 3-kinase delta syndrome	1 (1.8%)	10.1016/S2213-2600(22)00042-X
30	Wiskott-Aldrich syndrome	1 (1.8%)	10.1016/S2213-2600(22)00042-X
31	Immunodeficiency with short-limbed stature	1 (1.8%)	10.1016/S2213-2600(22)00042-X
32	Immunodeficiency following hereditary defective response to Epstein-Barr virus	1 (1.8%)	10.1016/S2213-2600(22)00042-X
33	Hyper-IgE syndromes	1 (1.8%)	10.1016/S2213-2600(22)00042-X
34	LFA-1 defect	1 (1.8%)	10.1016/S2213-2600(22)00042-X
35	Defects in the complement system	1 (1.8%)	10.1016/S2213-2600(22)00042-X
36	Sickle cell disease	1 (1.8%)	10.1093/infdis/jiaa786
37	Immune Reconstitution Inflammatory Syndrome	1 (1.8%)	10.1016/S2213-2600(22)00042-X
38	PML	1 (1.8%)	10.1093/brain/awac237

39	Hospitalisation for chemotherapy	1 (1.8%)	10.1093/infdis/jiaa786
40	Haematopoietic stem cell transplantation	1 (1.8%)	10.1016/j.lanepe.2022.100400
41	Acute and chronic graft-versus-host disease	1 (1.8%)	10.1016/S2213-2600(22)00042-X
42	SLD	1 (1.8%)	10.1016/j.cgh.2016.06.019
43	Cryoglobulinemia	1 (1.8%)	10.1016/S2213-2600(22)00042-X
44	Haematological neutropenia	1 (1.8%)	10.1093/infdis/jiaa786
45	Cyclophosphamide (L01AA01)	1 (1.8%)	10.1136/rmdopen-2022-002343
46	Janus-associated kinase inhibitors (L04AF)	1 (1.8%)	10.1007/s40744-020-00218-3
47	Monoclonal antibodies (L04AG)	1 (1.8%)	10.1007/s10620-021-07073-4
48	DHODH inhibitors (L04AK)	1 (1.8%)	10.1093/rheumatology/kez622
49	Inflammatory Bowel Disease	1 (1.8%)	10.1016/j.spinee.2017.11.007

ADA: Adenosine deaminase, AIDS: acquired immunodeficiency syndrome, DHODH: Dihydroorotate dehydrogenase, HIV: Human Immunodeficiency Virus, LFA-1: Lymphocyte function antigen-1, MHC: Major Histocompatibility Complex, PML: Progressive Multifocal Leukoencephalopathy, PNP: Purine nucleoside phosphorylase, SLD: Significant Liver Disease, TNF-α: Tumour necrosis factor alpha

Supplementary Table 4 (CSV file). *List of diagnostic and procedure codes included in the immunocompromise phenotype algorithm, mapped across different coding systems*[&*]

[&]As a precaution, we recommend opening this file in CSV format rather than in Excel. When opened directly in Excel, certain formats may be altered—for example, leading zeros may be removed, long codes may be converted to scientific notation, or dates may be reformatted as numbers.

*Every code in this file is tagged as *narrow*, *possible*, or *exclude* codes. *Narrow* tagged codes are intended to be highly specific to immunocompromise cases. *Possible* tagged codes are intended to provide a broader, more extensive capture of immunocompromise cases, but the degree of specificity for the concept of interest may vary by code. For a main outcome, we would recommend using *narrow* codes to decrease the risk of false positives. *Exclude* tagged codes identify codes that have been reviewed but decided not to be included to identify the concept.

Supplementary Table 5 (Excel file). *Immunocompromise Phenotype Algorithm Logic according to the ConcePTION CDM*

Supplementary Table 6. *List of the SAFETY-VAC study consortium members*

University Medical Center Utrecht (UMCU), Utrecht, The Netherlands	Dr. Carlos E. Durán,<br>Prof. Dr. Miriam Sturkenboom,<br>Dr. Judit Riera-Arnau,<br>Nicoletta Luxi
Universiteit Utrecht (UU), Utrecht, The Netherlands<br>CPRD data	Prof. Dr. Olaf Klungel,<br>Dr. Patrick Souverein
VAC4EU	Dr. Sima Mohammadi
Teamit Institute	Dr. Fabio Riefolo,<br>Dr. Irene Pazos
Agenzia Regionale di Sanitá Toscana (ARS)<br>Data Tuscany region	Dr. Rosa Gini,<br>Davide Messina,<br>Dr. Giuseppe Roberto
Società Servizi Telematici -Pedianet	Prof. Dr. Carlo Giaquinto,<br>Dr. Elisa Barbieri,<br>Luca Stona
Fundació Institut Universitari per a la Recerca a l'Atenció Primària de Salut Jordi Gol i Gurina (IDIAP JGol)	Dr. Felipe Villalobos,<br>Dr. Martín Solorzano,<br>Carlo Alberto Bissacco
Instituto Aragonés de Ciencias de la Salud (IACS)	Dr. Antonio Gimeno,<br>Dr. Beatriz Poblador,<br>Dr. Mercedes Aza,<br>Dr. Aida Moreno,<br>Alejandro Santos
Department of Clinical Epidemiology, Aarhus University and Aarhus University Hospital	Prof. Vera Ehrenstein,<br>Lise Skovgaard Svingel,<br>Benjamin Randeris Johannesen
Bordeaux PharmacoEpi platform (BPE) & ADERA	Dr. Cécile Droz-Perroteau,<br>Laure Carcaillon-Bentata,
University of Eastern Finland	Prof. Anna-Mija Tolppanen,<br>Prof. Sirpa Hartikainen,<br>Dr Thuan Vo,<br>Dr Anne Paakinaho,<br>Blair Rajamaki
University of Oslo (UiO), Norway	Prof. Dr. Hedvig Nordeng,

Norwegian linked registry data	Saeed Hayati,<br>Mahmoud Zidan
Foundation for the Promotion of Health and Biomedical Research of Valencia Region (FISABIO) – Valencia health system Integrated Database (VID)	Dr. Juan José Carreras Martínez,<br>Dr. Arantxa Urchueguía Fornes,<br>Elisa Correcher Martínez,<br>Dr. Javier Díez-Domingo
Spanish Agency on Medicines and Medical Devices (AEMPS) -BIFAP database	Dr. Mar Martin,<br>Dr. Patricia Garcia-Poza,<br>Dr. Airam de Burgos,<br>Belén Castillo-Cano,<br>Dr. Elisa Martín-Merino
